# Supplementary material for: Geography is a stronger predictor of diversification of monogenean parasites (Platyhelminthes) than host relatedness in characin fishes of Middle America
Source: PLoS One. 2025 Apr 29;20(4):e0316974. doi: 10.1371/journal.pone.0316974 (PMC12040092; doi:10.1371/journal.pone.0316974)

S1 Fig. Cophylogenetic plot showing associations between characin fish hosts and their monogenean parasites.
Phylogenetic trees are ML trees in Figs 1B and 2A. Line colors correspond to areas of endemism.

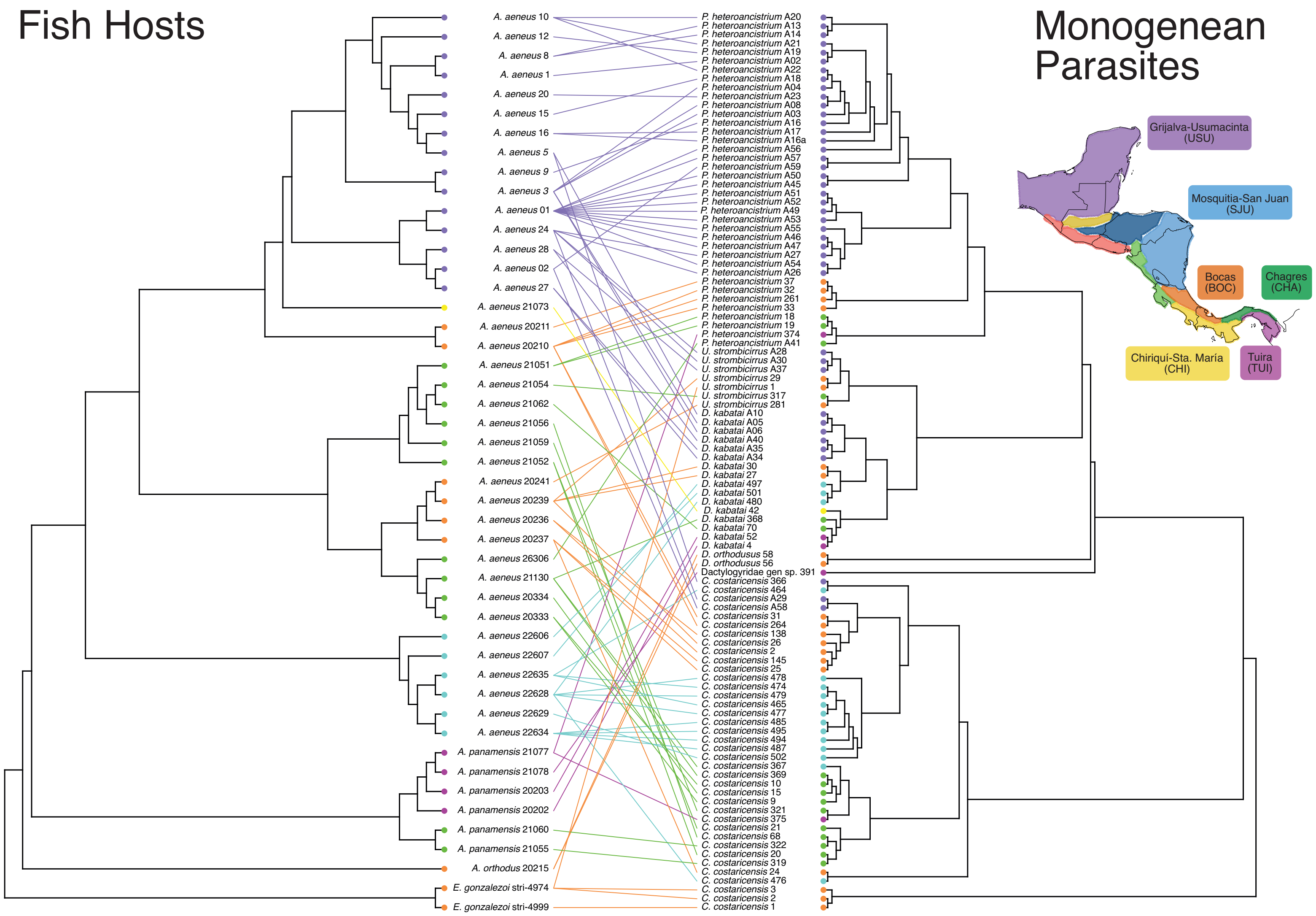

Supplement: S1 Fig — Phylogenetic trees are ML trees in Figs 1B and 2A. Line colors correspond to areas of endemism. (PDF) [file pone.0316974.s004.pdf]
